# Successful Nonsurgical Resolution of a Large Periapical Lesion With Pre‐Existing Sealer Extrusion and Adjacent External Inflammatory Root Resorption: A Case Report

**DOI:** 10.1155/crid/6413120

**Published:** 2026-10-12

**Authors:** Btissam Seffahi, Nisrine Bassim, Faïza Abdallaoui

**Affiliations:** ^1^ Department of Conservative Dentistry and Endodontics Faculty of Dental Medicine Mohammed V University Rabat Morocco um5s.ac.ma

**Keywords:** calcium hydroxide, endodontics, periapical periodontitis, root canal therapy, tooth resorption

## Abstract

The primary aim of root canal treatment is to eliminate intracanal infection, achieve an apical and coronal seal, and create conditions that allow repair of the periapical tissues. In root‐filled teeth, persistent apical periodontitis may be associated with extensive periapical lesions, external inflammatory root resorption affecting the treated or adjacent teeth, and extrusion of endodontic materials. These complications may lead clinicians to consider surgical approaches. However, when canals are adequately cleaned, disinfected, and hermetically obturated, favorable healing may occur following nonsurgical retreatment alone. This case report describes the nonsurgical management of a large periapical lesion associated with a previously treated maxillary lateral incisor. The lesion was complicated by pre‐existing sealer extrusion and by external apical inflammatory resorption of the adjacent maxillary central incisor. Repeated calcium hydroxide intracanal medication and obturation were performed, followed by functional coronal restoration. Ongoing radiographic healing and clinical stability were observed at 20 months of follow‐up, supporting an orthograde, conservative approach in similar clinical situations.

Clinical Relevance

Large periapical lesions associated with root‐filled teeth may resolve following nonsurgical endodontic retreatment when effective canal decontamination and an adequate coronal seal are achieved. Conservative management with structured clinical‐radiographic follow‐up may be appropriate in selected cases, even in the presence of extruded sealer and associated external inflammatory root resorption.

## 1. Introduction

Apical periodontitis is a chronic inflammatory disease of the periapical tissues caused by microorganisms of endodontic origin and their byproducts, leading to resorption of alveolar bone and its replacement by inflammatory tissue [1–3]. Epidemiological data indicate that apical periodontitis affects a substantial proportion of root‐filled teeth, with a prevalence reported as high as 39%, highlighting the burden of treatment failure and the need to identify and manage persistent endodontic infection [3]. Recognized causes of posttreatment apical periodontitis include insufficient irrigation and instrumentation, incomplete obturation, coronal microleakage, and persistent intraradicular or extraradicular biofilms, as well as procedural complications such as sealer extrusion beyond the apex [1, 4, 5]. External inflammatory root resorption represents a further challenge when present in association with apical periodontitis. It develops when an infected root canal system communicates with damaged external root surfaces or periradicular tissues, allowing bacteria and/or their endotoxins to perpetuate inflammation and activate clastic cells that resorb root structure and adjacent bone [6]. In such cases, the primary therapeutic objective is to eliminate the intracanal infection and interrupt the resorptive process. Calcium hydroxide remains a widely used intracanal medicament for teeth with large periapical lesions and inflammatory root resorption, owing to its alkaline pH, antibacterial and antiresorptive effects, neutralization of endotoxins, and stimulation of mineralized tissue formation [7, 8]. For teeth with posttreatment apical periodontitis, both nonsurgical retreatment and surgical endodontic procedures are valid options, and current evidence does not demonstrate clear superiority of one approach over the other in terms of periapical healing [5, 9].

The simultaneous occurrence of a large periapical lesion, apically extruded sealer, and external inflammatory resorption of an adjacent, previously untreated tooth is uncommon and is frequently regarded as an indication for surgical intervention. The present case report describes the successful nonsurgical management of a previously treated maxillary lateral incisor with concomitant external apical inflammatory resorption of the adjacent central incisor, using calcium hydroxide as an intracanal medicament and lateral condensation of gutta‐percha with a zinc oxide–eugenol (ZOE) sealer, with a 20‐month follow‐up, supporting an orthograde approach in comparable situations.

## 2. Case Presentation

A 50‐year‐old woman with no notable medical history was referred to the Department of Conservative Dentistry and Endodontics at our institute to treat a large radiographic radiolucency extending apically from Tooth #13 to #21. Her chief complaint was a mild tenderness in the upper labial vestibule and subnasal region. The patient reported no spontaneous pain or any previous dental trauma. Dental history included nonsurgical root canal treatment performed 8 years earlier for Tooth #12, and a restoration on Tooth #13 around the same time; no previous dental interventions were done for Teeth #11 and #21. Extraoral examination showed no facial swelling. An intraoral examination revealed the presence of defective resin‐composite coronal restorations with overhanging mesial and distal margins on #12 with a satisfactory palatal restoration, and a satisfactory resin‐composite mesial restoration on #13. No signs of periodontal involvement were detected, as confirmed by mobility tests and periodontal probing. Thermal and electric pulp testing was performed for all upper anterior teeth, and all responded normally except for Teeth #12 and #11, which were nonresponsive. No pain was reported on percussion and palpation of all examined teeth.

An external maxillofacial multidetector computed tomography (MDCT) had been obtained by the referring maxillofacial surgeon to investigate the subnasal/vestibular discomfort. The MDCT (Figure 1) showed a well‐defined periapical radiolucency in the anterior maxilla in relation to the apex of #12, with thinning and possible focal dehiscence of the labial cortical plate and an intact palatal cortex; the floor of the nasal cavity appeared preserved, with no evident sinus or nasal communication. Multiplanar reconstructions from this external medical scan were used solely to assess the macrotopography of the main lesion and did not contribute to the 3D evaluation of Tooth #11′s root resorption. A panoramic overview (Figure 2) showed a well‐defined periapical radiolucency in the right anterior maxilla, centered on the apex of #12 and extending from #13 to #21, with no displacement of adjacent anatomical structures. Standardized periapical radiographs (Figure 3) captured only the central portion of the lesion around the apices of #13, #12, and #11; and its full extent could not be appreciated on the periapical views. Tooth #12 had a radiopaque filling material extending in the root canal space with an extruded sealer beyond the apex. Tooth #11 showed external apical root resorption with a radiographically closed apex. Using the periapical index (PAI), the baseline periapical status was scored as PAI 5 for both #12 and #11 [10].

**Figure 1:**
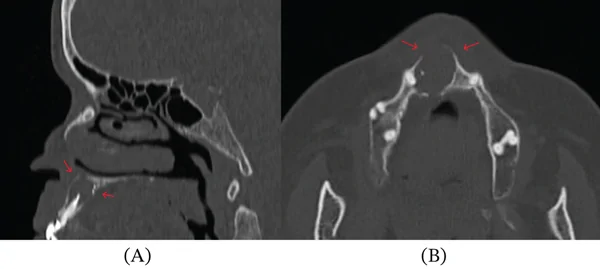
(A–B) Preoperative maxillofacial MDCT showing sagittal and axial sections of the anterior maxilla. Red arrows indicate the borders of the periapical lesion and the labial cortical thinning. Date: February 20, 2024.

**Figure 2:**
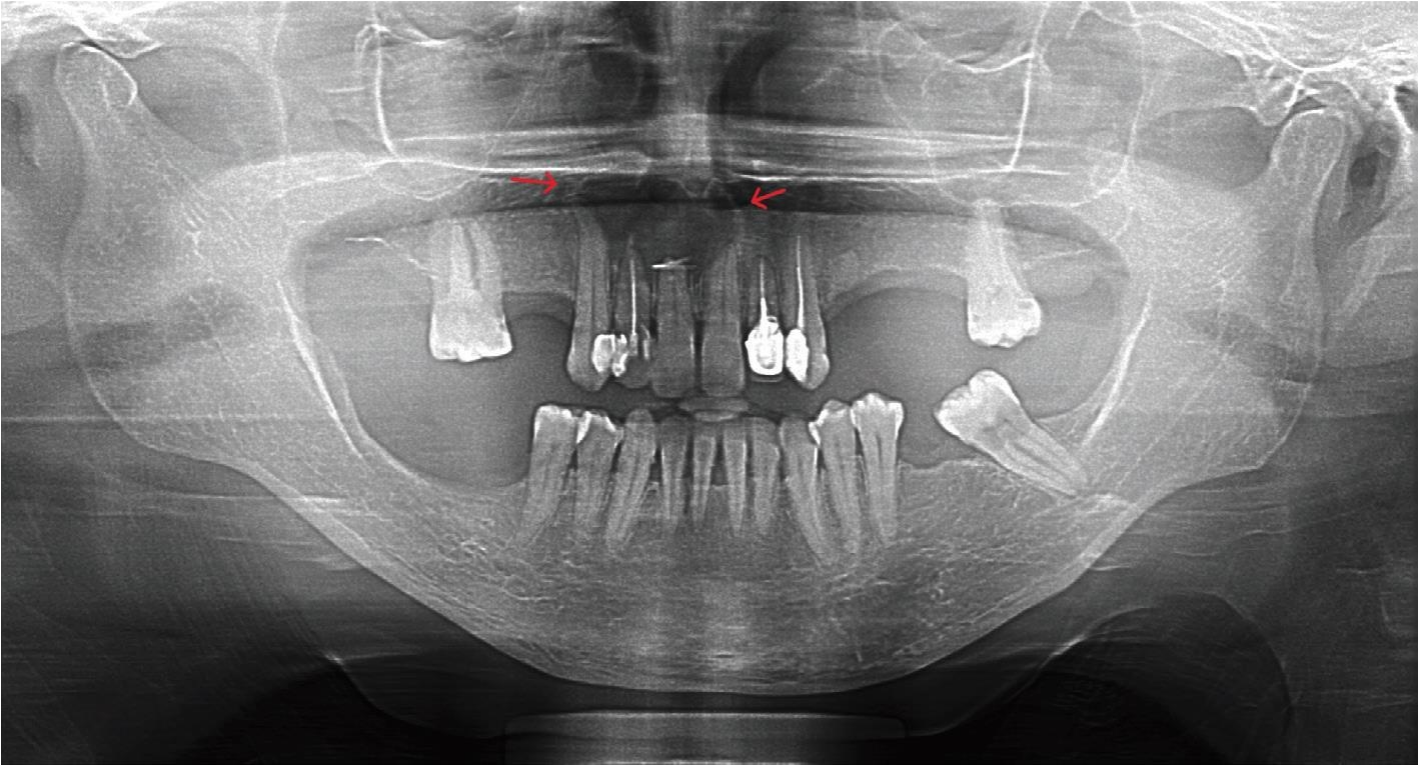
Preoperative panoramic radiograph. Red arrows indicate the superior extent of the periapical radiolucency in the right anterior maxilla. Date: February 27, 2024.

**Figure 3:**
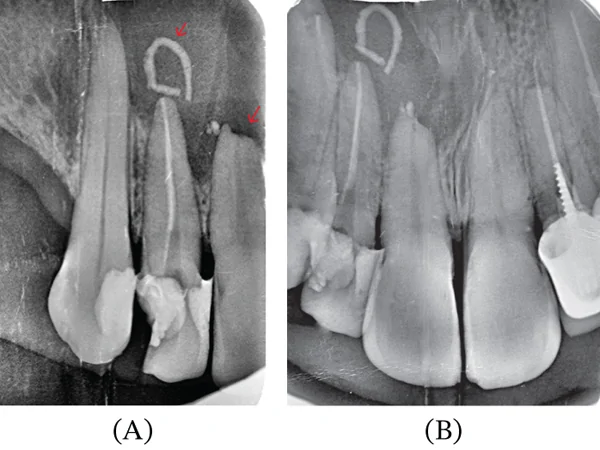
(A–B) Preoperative periapical radiographs taken at different horizontal angulations. In (A), red arrows indicate the sealer extruded beyond the apex of #12 and the external apical resorption of #11; (B) is a second angulation centered on #11 and #21. Minor image editing was performed to reduce linear film artifacts. Date: February 27, 2024.

Based on the clinical and radiographic findings, Tooth #12 was diagnosed as a previously treated tooth with chronic apical periodontitis, and Tooth #11 as pulpal necrosis with chronic apical periodontitis and external apical inflammatory root resorption. Given the considerable size of the lesion and the presence of external inflammatory resorption on the adjacent tooth, both a periapical granuloma and a radicular cyst were considered plausible. Larger, long‐standing periapical lesions carry a higher probability of being cystic, although radiographic features alone cannot reliably distinguish the two entities. A foreign‐body reaction related to the apically extruded sealer of #12 was also considered, given its potential to sustain periapical inflammation. As no biopsy or histological examination was performed, a definitive histopathological diagnosis could not be established, and the lesion was managed on the basis of its clinical and radiographic presentation.

One week after baseline imaging, nonsurgical endodontic treatment was initiated. Treatment was performed under rubber dam isolation after written informed consent was obtained from the patient. Tooth #12 was addressed first; defective restorations were removed and a pre‐endodontic restoration was placed with a resin‐modified glass ionomer cement (Riva Light Cure HV, SDI Limited, Bayswater, Victoria, Australia) to restore missing walls and secure isolation. Access cavities were prepared in #12 and #11 with a round diamond bur (ISO 010; Edenta AG, Schaan, Liechtenstein) and a safe‐end Endo‐Z bur (Dentsply Sirona, Ballaigues, Switzerland). On access, the pulp chamber of #11 contained necrotic tissue, confirming pulp necrosis. In #12, the previous root filling material was removed using ProTaper Universal Retreatment files (Dentsply Sirona, Ballaigues, Switzerland). Canal negotiation for both teeth was performed with #10 and #15 K files, and working length was established with an electronic apex locator (iPex II, NSK Nakanishi Inc., Tokyo, Japan) and confirmed radiographically. Cleaning and shaping of #12 and #11 were completed with the ProTaper Gold rotary system to F2 (Dentsply Sirona Ballaigues, Switzerland). Apical patency was maintained throughout instrumentation with a size 15 K‐file. The canals were prepared to a master apical size of F2 (ISO 25), confirmed by apical gauging, with the apical foramen kept at its natural diameter and not enlarged further. Irrigation during instrumentation used a 5% sodium hypochlorite (NaOCl) solution (MAARC Dental, India), approximately 2 mL delivered with a hypodermic needle after each instrument. At this stage, there was persistent serous exudate; therefore, an intracanal medicament consisting of a thick calcium hydroxide paste (Ca(OH)_2_ powder: Calcident, Omega Medic, Casablanca, Morocco) mixed chairside with vasoconstrictor‐free local anesthetic (Scandonest 3% mepivacaine 30 mg/mL, Septodont, France) was placed with pluggers, and the access cavities were sealed with a temporary ZOE cement prepared chairside (Oxydia ZnO powder, Vial Medical, Casablanca, Morocco; eugenol, Omega Medic, Casablanca, Morocco). No adjunctive systemic medications were prescribed.

The Ca(OH)_2_ dressing was renewed at two subsequent visits at 14‐day intervals (approximately 2 and 4 weeks after initiation) until no further exudate was observed and the canals were dry. Between the second and third visits, Tooth #12 fractured coronally, and a glass‐ionomer dome was placed with GC Fuji II (capsule) (GC Corporation, Tokyo, Japan) to maintain a coronal seal and isolation between appointments.

At approximately 6 weeks from initiation, the temporary dressings were removed. Copious irrigation was performed with 5% NaOCl, followed by 17% EDTA (Produits Dentaires SA, Vevey, Switzerland); the NaOCl and EDTA were energized with a size 25 EndoActivator (Dentsply Sirona, Charlotte, NC) for 1 min. A ProTaper Universal F2 gutta‐percha master cone (Dentsply Sirona) matched to the preparation was then fitted to the working lengths with confirmed tug‐back in both teeth; in Tooth #11, the cone was shortened by 0.5 mm to achieve adequate tug‐back. A final rinse with 0.9% sodium chloride (Laprophan, Casablanca, Morocco) was performed, and after drying with paper points, the canals were obturated by lateral condensation of gutta‐percha points (Dentsply Maillefer, Ballaigues, Switzerland) with a ZOE sealer (Oxydia ZnO powder, Vial Medical, Casablanca, Morocco; eugenol, Frey&Lau GmbH, Germany). A minor apical extrusion (puff) of sealer was noted in both teeth on the postobturation radiograph. Tooth #11 received a resin‐composite restoration (CharmFil Plus, Dentkist Co. Ltd., Republic of Korea). Tooth #12 was restored with a provisional prosthetic resin crown to restore function and esthetics and to support periapical healing. The full treatment sequence is summarized in Table 1.

**Table 1:** Chronological treatment timeline.

Timepoint	Calendar date	Key procedure
Baseline	20/02/2024	Diagnosis and imaging; baseline PAI 5 for #12 and #11
Treatment start	27/02/2024	Retreatment of #12 and treatment of #11: cleaning and shaping; first Ca(OH)_2_ dressing
2 weeks	12/03/2024	Ca(OH)_2_ renewed
4 weeks	26/03/2024	Ca(OH)_2_ renewed (interim GC Fuji II dome after #12 coronal fracture)
6 weeks	09/04/2024	Final irrigation and obturation; restoration of #11 and #12
20 months	14/10/2025	Asymptomatic; PAI #12 5 → 4 (ongoing healing), #11 5 → 2; residual radiolucency at #12; CBCT planned at 2 years

Follow‐up at 20 months showed that the patient was asymptomatic, and Teeth #11 and #12 responded normally to percussion and finger palpation tests. Panoramic and periapical radiographs (Figures 4 and 5) showed a reduction of the radiolucency and significant bone formation, with a residual radiolucency persisting at the apex of #12. Using the PAI, the score for #12 decreased from 5 to 4 and that for #11 from 5 to 2. These findings are consistent with incomplete/ongoing radiographic healing for Tooth #12, near‐complete periapical healing for Tooth #11, and overall radiographic stabilization of the external apical inflammatory resorption [10]. Tooth #12 remained restored with the provisional crown, with definitive prosthetic rehabilitation planned once healing is confirmed. A limited field‐of‐view CBCT is planned at 2 years to confirm resolution of the lesion and stabilization of the resorptive defect on #11.

**Figure 4:**
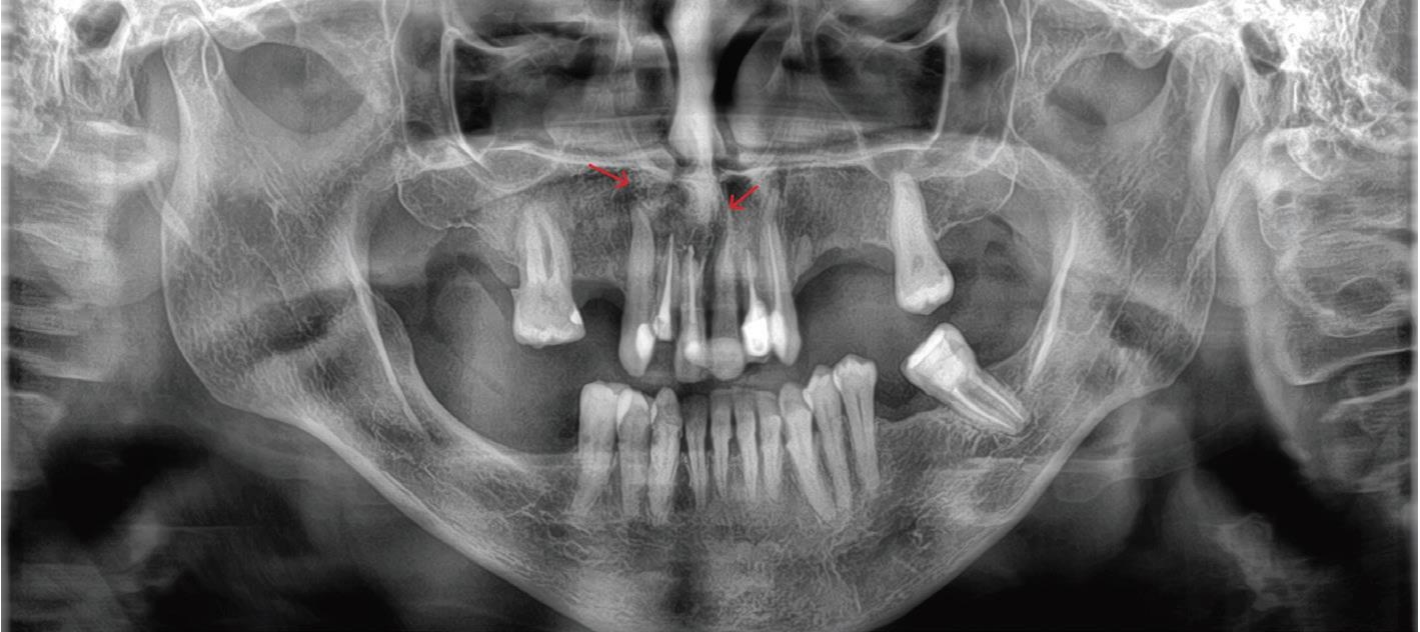
Panoramic radiograph at 20‐month follow‐up. Red arrows indicate the region of previous radiolucency, now showing bone formation. Date: October 14, 2025.

**Figure 5:**
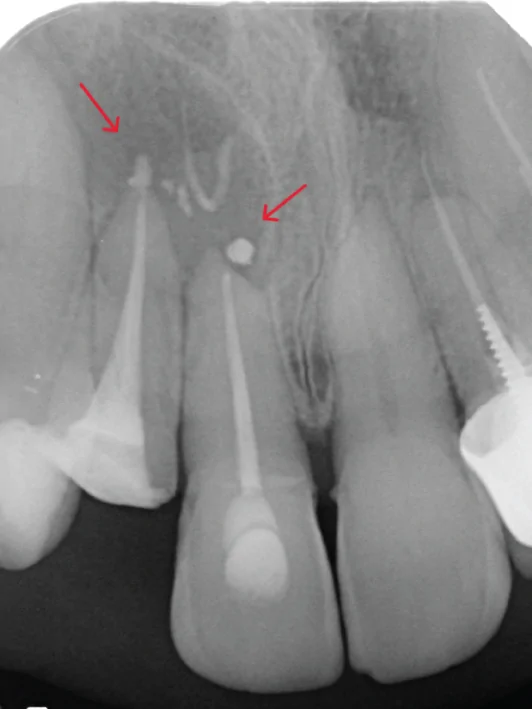
Standardized periapical radiograph at 20‐month follow‐up, showing reduction of the periapical radiolucency with bone formation and a residual radiolucency at the apex of #12. Red arrows indicate the obturation sealer puffs at the apices of #11 and #12; extruded sealer from the previous treatment also remains between the apices of #11 and #12. Date: October 14, 2025.

## 3. Discussion

This case combined a large periapical lesion in a previously root‐filled maxillary lateral incisor with external apical inflammatory resorption of the adjacent central incisor. The previous treatment of Tooth #12 showed incomplete root‐filling, defective coronal restorations, and extrusion of sealer beyond the apex—features commonly associated with persistent apical periodontitis and endodontic treatment failure [1, 4, 5, 11]. As no clear evidence of superiority has been demonstrated between nonsurgical and surgical approaches for the management of apical periodontitis in root‐filled teeth [5, 9], nonsurgical root canal retreatment of Tooth #12 and root canal treatment of Tooth #11 were selected as conservative strategies to eliminate the endodontic infection and manage both the periapical lesion and the resorptive process.

A particularly instructive feature of this case is the external apical inflammatory resorption of Tooth #11, an adjacent, previously untreated tooth. External inflammatory resorption is initiated by damage to the protective precementum layer of the external root surface and is sustained by microorganisms and their byproducts diffusing from an infected, necrotic pulp. While classically associated with luxation trauma, it also occurs in the majority of infected teeth with chronic apical periodontitis [6, 12]. In this case, pulp necrosis of Tooth #11 was confirmed both by its lack of response to thermal and electric sensibility testing and by the necrotic pulp tissue found on access opening, indicating an infected root canal system that most plausibly sustained the resorptive process. Although no dental trauma was reported, a previous, unrecognized luxation injury cannot be excluded as the initial cause of the cemental damage, and the large adjacent periapical lesion of #12 may have added to the periradicular inflammatory burden. Eliminating the intracanal infection in both teeth removed the stimulus for clastic activity, consistent with the improvement in the periapical status of #11, whose PAI score decreased from 5 to 2, and the radiographic stabilization of its resorptive defect. This highlights the importance of assessing, and where indicated, treating nonvital teeth associated with a periapical lesion, since controlling the root canal infection is central to arresting external inflammatory resorption.

Calcium hydroxide was selected as the intracanal medicament because it remains a widely used and reliable agent for disinfecting root canals with large periapical lesions and for preventing and treating inflammatory root resorption due to its alkaline pH, antibacterial and antiresorptive effects, neutralization of endotoxins, and stimulation of mineralized tissue formation [7, 8]. Here, Ca(OH)_2_ powder was mixed chairside with a dental local anesthetic solution, as a liquid vehicle is required for hydroxyl ion release. Although sterile water or saline is commonly used, local anesthetic solutions have been reported as suitable aqueous carriers, and their acidic pH has minimal effect because Ca(OH)_2_ is a strong base [7]. The dressing was renewed every 14 days, which lies within the recommended 7‐ to 14‐day interval for Ca(OH)_2_ application, and maintained for a total of 6 weeks. Although this slightly exceeds the 4‐week period at which a possible reduction in dentine fracture resistance has been reported, it was chosen as a clinically acceptable compromise to ensure sustained disinfection and control of external inflammatory root resorption in the presence of a large periapical lesion [13, 14].

Cold lateral compaction with gutta‐percha and a ZOE sealer was used for obturation. In Tooth #11, the radiographically closed apex made this technique appropriate without the need for an artificial apical barrier. Gutta‐percha remains the standard core material, and ZOE‐based sealers are still widely employed in routine endodontic practice [15]. A recent systematic review reported better periapical healing with bioceramic and other bioactive sealers compared with ZOE‐based sealers [16]. In the present case, the favorable evolution nevertheless suggests that, when combined with adequate chemomechanical preparation and a satisfactory coronal seal, conventional ZOE‐based obturation can still support successful periapical healing.

The coronal restoration also plays a key role in the outcome of endodontically treated teeth. Teeth with adequate coronal restorations show a lower prevalence of apical periodontitis and better periapical healing than those with defective or absent restorations [17, 18]. By preventing coronal microleakage and restoring functional loading, a well‐adapted provisional crown can support periapical bone remodeling and lesion resolution [19]. For this patient, the provisional prosthetic resin crown on Tooth #12 was intended to provide both a coronal seal and functional stimulation to favor healing of the large periapical lesion. Radiographic healing of periapical lesions is, however, a slow, progressive process. Clinical follow‐up studies indicate that most lesions that eventually heal do so within the first 2 years after treatment, but the proportion of healed cases continues to increase when observation is extended up to 4 years. Complete radiographic resolution has been reported even later in some cases [20–22].

Extraradicular foreign materials, such as apically extruded sealers or remnants of intracanal medicaments, have been reported as possible causes of persistent apical lesions, because they can maintain a low‐grade foreign‐body reaction and prevent complete resolution until the material is removed [1, 4, 5, 11]. Consistent with this, a systematic review with meta‐analysis found sealer extrusion to be associated with a modestly lower success rate [23]. A more recent study using a within‐tooth design, comparing extruded and nonextruded roots in the same patients, found that extrusion was associated with delayed rather than failed healing in teeth with pre‐existing periapical lesions. Longer follow‐up was recommended [24]. Nevertheless, clinical and histological studies suggest that periapical repair depends mainly on effective control of intracanal infection rather than complete removal of all material from the periapical tissues [1, 25], and that extruded material, ZOE sealer in particular, is progressively phagocytosed and resorbed from the periradicular tissues [26], as would be expected for the minor puff extruded during the present obturation. Extruded sealer may therefore slow healing but does not necessarily prevent it when disinfection is adequate and the host response is favorable. In this patient, the marked reduction in lesion size and the decrease in PAI scores from 5 to 4 for Tooth #12 and from 5 to 2 for Tooth #11 at 20 months, despite the persistence of a sealer residue beyond the apex of Tooth #12, are therefore compatible with an ongoing healing process and support continued clinical and radiographic monitoring.

The persistence of a small residual radiolucency at the apex of Tooth #12, in contrast to the near‐complete healing at Tooth #11, warrants cautious interpretation. It may represent apical scar tissue (a fibrous, radiolucent but nonpathological repair), incomplete slow osseous healing still ongoing at 20 months, or low‐grade persistent inflammation sustained by the extruded sealer. The absence of symptoms and the favorable radiographic trend favor continued healing or scar formation rather than active disease, although only extended follow‐up, supported by the planned CBCT, can reliably distinguish these possibilities.

One of the limitations of this case is that diagnosis and treatment planning were based on two‐dimensional radiographs (panoramic and standardized periapical views). According to the European Society of Endodontology′s position statement, a limited field of view (FOV) CBCT may be indicated in cases of root resorption that appear amenable to treatment, as it overcomes superposition and geometric distortion and allows a more accurate three‐dimensional assessment of lesion extent and root morphology [27]. CBCT was not obtained at the time of treatment because the patient declined it for financial reasons after having already undergone a medical CT scan. Furthermore, the pre‐existing medical MDCT lacked the spatial resolution required for fine‐grained 3D tracking of the root resorption on Tooth #11. Quantitative dimensions of the lesion were not obtained, as the MDCT had been performed at an external center and the original dataset was no longer accessible for measurement. A limited FOV CBCT is planned at 2 years to provide a three‐dimensional evaluation of the resorptive defect and long‐term healing of the lesion. Further limitations should be acknowledged. First, no histological examination of the lesion was performed, so its precise nature (e.g., radicular cyst versus granuloma) could not be confirmed. Second, a conventional ZOE sealer was used rather than a bioceramic material; although some evidence suggests better periapical healing with bioceramic sealers [16], a randomized clinical trial and a systematic review with meta‐analysis directly comparing calcium silicate–based and ZOE sealers reported no significant difference in treatment outcome [28, 29], and the favorable evolution observed in this case is consistent with these findings. Finally, being a single case report, these findings cannot be generalized and require confirmation in larger, controlled studies.

## 4. Conclusion

In the present case report, a nonsurgical endodontic approach with careful cleaning, decontamination, and filling of the root canal system, together with calcium hydroxide as an intracanal medicament and obturation with gutta‐percha and a ZOE sealer, resulted in favorable healing of a large periapical lesion associated with external inflammatory root resorption. This supports the view that, when the tooth is restorable and adequate disinfection can be achieved, endodontic therapy without surgery should, whenever possible, be attempted first for the management of extensive periapical lesions [5], and that conventional materials can still provide predictable outcomes when they represent the only available option. Regular clinical and radiographic follow‐up remains necessary to confirm lesion resolution and the long‐term stability of healing. As this is a single case, these findings cannot be generalized and should be interpreted in the context of the existing evidence on nonsurgical management of extensive periapical lesions. Further clinical studies are needed to clarify the prognosis and indications for nonsurgical (re)treatment in similar situations.

## Author Contributions

Clinical management and data acquisition: B.S. (supervised by N.B.). Manuscript drafting: B.S. Manuscript revision and editing: B.S. and N.B. Critical revision of the manuscript: F.A.

## Funding

No funding was received for this manuscript.

## Disclosure

All authors approved the final manuscript. The authors reviewed and revised the manuscript and take full responsibility for its content.

## Consent

Written informed consent was obtained from the patient for publication of anonymized clinical data and images.

## Conflicts of Interest

The authors declare no conflicts of interest.

## Data Availability

Data sharing is not applicable to this article as no datasets were generated or analyzed during the current study.
